# Serum proteins are extracted along with monolayer cells in plasticware and interfere with protein analysis

**DOI:** 10.14440/jbm.2016.129

**Published:** 2016-08-13

**Authors:** Xin Hong, Yuling Meng, Steven N. Kalkanis

**Affiliations:** Department of Neurosurgery, Henry Ford Health System, Detroit, Michigan 48202, USA

**Keywords:** bovine serum protein, cell culture, protein adsorption, protein extraction, serum albumin

## Abstract

Washing and lysing monolayer cells directly from cell culture plasticware is a commonly used method for protein extraction. We found that multiple protein bands were enriched in samples with low cell numbers from the 6-well plate cultures. These proteins contributed to the overestimation of cell proteins and led to the uneven protein loading in Western blotting analysis. In Coomassie blue stained SDS-PAGE gels, the main enriched protein band is about 69 kDa and it makes up 13.6% of total protein from 10^4^ U251n cells. Analyzed by mass spectrometry, we identified two of the enriched proteins: bovine serum albumin and bovine serum transferrin. We further observed that serum proteins could be extracted from other cell culture plates, dishes and flasks even after washing the cells 3 times with PBS. A total of 2.3 mg of protein was collected from a single well of the 6-well plate. A trace amount of the protein band was still visible after washing the cells 5 times with PBS. Thus, serum proteins should be considered if extracting proteins from plasticware, especially for samples with low cell numbers.

## INTRODUCTION

Lysing cultured cells is a common method for extracting proteins. To this end, cells can be dissociated by trypsin-EDTA, centrifuged to pellet cells, and then proteins are extracted from the cell pellet. Another option is to lyse monolayer cells directly from cell culture plasticware. To avoid protein degradation in the process of cell detachment, directly lysing monolayer cells is required for studies targeting sensitive proteins (*i.e.*, cell-signaling proteins). In this process, monolayers are washed with ice-cold phosphate-buffered saline (PBS) 2 to 3 times, and lysis buffer is then added to extract cell proteins.

Fetal bovine serum (FBS) is the most widely used supplement for the culture of normal and cancer cells. FBS, prepared as 10% (v/v) in medium, is necessary for cell growth *in vitro*. When preparing cell lysate from monolayers, most serum proteins are removed and the residual FBS is not a problem for samples containing a large amount of cell protein. However, if low cell numbers or low protein samples are to be extracted, the residual FBS proteins may be problematic. We found that serum proteins in cell culture medium can be adsorbed by cell culture plasticware and cannot be entirely removed by a general washing step. The residual serum proteins may cause overestimation of total cell protein and interfere with subsequent protein analysis.

## MATERIALS AND METHODS

### Materials

Gibco qualified fetal bovine serum (cat. # 10437), high glucose Dulbecco’s Modified Eagle Medium (DMEM) (cat. # 12800), Trypsin-EDTA (0.25%, catalog 25200), PBS (cat. # 10010) and antibiotic (cat. # 15240) were obtained from Thermo Fisher Scientific, Inc. (Grand Island, NY). Cell culture flasks (T25, cat. # 430639 and T75, cat. # 430641), 24-well plates (cat. # 3524), 6-well plates (cat. # 3516) and 100 mm × 20 mm dishes (cat. # 430167) were ordered from Corning Incorporated (Corning, NY).

### Cell culture

LN229, T98G, U251n and U87 glioblastoma cell lines were obtained from American Type Culture Collection (ATCC). Cells were cultured with DMEM containing 10% (v/v) FBS, 100 IU/ml penicillin, 100 μg/ml streptomycin and 25 µg/mL of fungizone in a 37°C incubator supplied with 5% CO_2_.

### Cell lysate preparation

Cell culture media were carefully removed from culture plasticware, and glioma monolayers were washed three times with ice-cold PBS. Radioimmunoprecipitation assay buffer (RIPA) (50 mM Tris pH 7.4, 250 mM NaCl, 5 mM EDTA, 1% NP‑40, 0.1% SDS, and 0.5% sodium deoxycholate) containing 1% protease inhibitor cocktail (Calbiochem, San Diego, CA) was added directly onto cells. A pipette was used to wash the area where cells were growing, and cell lysate was passed through the pipette 20 times to form homogeneous lysate. The lysate was then transferred to a 1.5-ml microcentrifuge tube. The resulting mixture was centrifuged at 14,000 g for 10 min at 4°C . The supernatant was removed from the cellular debris and saved for protein quantification. Protein concentration was determined using the BCA protein assay kit (Pierce, Rockford, IL).

### SDS-PAGE and Coomassie blue staining

The Mini-PROTEAN Tetra Vertical Electrophoresis System (Bio-Rad, cat. # 1658000FC) was used to hand cast polyacrylamide gels. Forty percent acrylamide/bis solution (37.5:1, 2.6% crosslinker) and other reagents for gel casting and running were purchased from Bio-Rad Laboratories, Inc. (Hercules, CA). Protein samples were denatured with Laemmli buffer [1] and applied to 8% polyacrylamide gels for electrophoresis.

Following electrophoresis, the gel was placed in a solution of 40% methanol and 10% acetic acid containing 0.25% Coomassie Brilliant Blue R-250 (Fisher Scientific, Inc., cat. # BP101, Fair Lawn, NJ) for 2 h. The gel was de-stained with several changes of distilled water overnight until the background was transparent. A stain-free method was also used to visualize proteins. 2,2,2-trichloroethanol (TCE) (Sigma, St. Louis, MO) was added to the separating gels at a concentration of 0.5%. Proteins were visualized under ultraviolet (UV) light for 5 min [2].

### Western blotting analysis

Total protein of 8−10 μg was denatured and subjected to SDS-PAGE, transferred to polyvinylidene fluoride (PVDF) membrane, and probed with antibodies, followed by HRP-conjugated secondary antibodies. Specific proteins were detected by ECL Western Blotting Detection Reagents (GE Healthcare Biosciences). Bovine serum albumin antibody was purchased from Thermoscientific (cat. # A11133). β-actin antibody was obtained from Sigma. The antibody against Nestin was purchased from Millipore (Temecula, CA). Antibodies against Glyceraldehyde 3-phosphate dehydrogenase (GAPDH), protein kinase B (AKT), extracellular signal-regulated kinase (ERK) and Focal Adhesion Kinase (FAK) were obtained from Cell Signaling Technology (Danvers, MA). For quantification of protein levels, densitometric analysis was carried out using ImageJ software (National Institutes of Health, Baltimore, MD).

### Mass spectrometry

Proteins from 1 × 10^4^ LN229 cells in 6-well plates were applied to SDS-PAGE gel. The gel was stained by Coomassie brilliant blue, and 2 main bands were excised. The gel pieces were washed with water, 25 mM ammonium bicarbonate (NH_4_HCO_3_), and then washed with 50% acetonitrile (ACN) for 15 min each. The liquid was removed and the gel pieces were dehydrated in 100% ACN for 5 min. Once the liquid was removed, the gel pieces were rehydrated in 50 mM NH_4_HCO_3_. After 5 min, an equal volume of 100% ACN was added and the gel pieces were left to incubate in the solution for 15 min. All liquid was then removed and the gel pieces were dehydrated once again in 100% ACN for 5 min. Once the liquid was removed after the incubation, the gel pieces were speed vacuumed dry for 5 min. Protein samples were then reduced with 5 mM dithiothreitol (DTT) and 50 mM NH_4_HCO_3_, alkylated with 15 mM iodoacetamide (IAA) and 50 mM NH_4_HCO_3_, and then digested overnight with sequencing-grade trypsin (Promega) in 40 mM NH_4_HCO_3_, 1 mM calcium chloride (CaCl_2_), and 0.01% Protease Max (Promega, Madison, WI). Following digestion, peptides were extracted from the gel plugs using 1% formic acid (FA). The free peptides were then speed vacuumed dry and solubilized in 0.1% FA.

The peptides were separated by reverse-phase chromatography (Acclaim PepMap100 C18 column, Thermo Fisher Scientific, Inc.), followed by ionization with the Nanospray Flex Ion Source (Thermo Fisher Scientific, Inc.), and introduced into an Orbitrap Fusion™ mass spectrometer (Thermo Fisher Scientific, Inc., FSN 10116).

### Proteome analysis

Abundant species were fragmented with high energy collision-induced dissociation (HCID). Data analysis was performed using Proteome Discoverer 1.4 (Thermo Fisher Scientific, Inc.) which incorporated the Mascot (Matrix Science) and Sequest algorithms (Thermo Fisher Scientific, Inc.). The Uniprot_Hum_Compl_20150826 and Uniprot_Bov_Compl_20140624 databases were searched for human and bovine protein sequences and a reverse decoy protein database was run simultaneously for false discovery rate (FDR) determination. Secondary analysis was performed using Scaffold 4.4.5 (Proteome Software). Minimum protein identification probability was set at <= 1.0% FDR with 2 unique peptides at <= 0.1% FDR minimum peptide identification probability.

## RESULTS AND DISCUSSION

**Figure 1:**
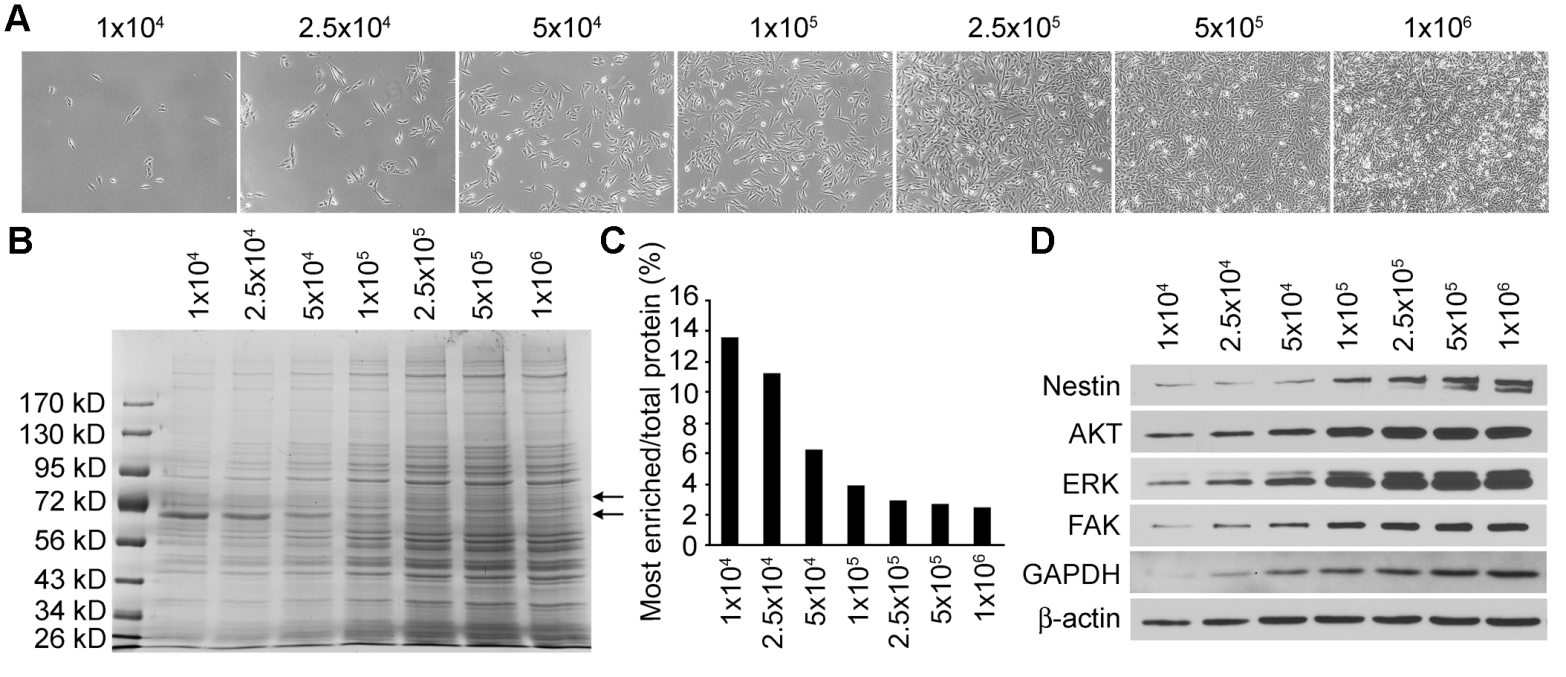
**Enrichment of multiple proteins in extractions with low cell numbers**. **A**. Photograph of increasing number of U251n cells in 6-well plate for 24 h (10 ×). **B**. U251n cell proteins were extracted from monolayers in 6-well plates and 8 µg of total proteins were loaded into 8% SDS-PAGE gels. The gels were stained with Coomassie brilliant blue. Arrows indicate the bands enriched at low cell number samples. **C**. Densitometric analysis of the most enriched band (lower arrow). Results were shown as the percentage of the band to total protein of each lane. **D**. Western blotting analysis of several proteins using the U251n cell samples. These experiments were repeated multiple times and a typical result is shown here.

When performing cell-density related protein-expression analysis using glioma cells (**Fig. 1A**), we found that multiple protein bands were enriched in low cell number samples in a SDS-PAGE gel. Two of the most highly enriched protein bands had approximate molecular weights of 69 kDa and 75 kDa (**Fig. 1B**). The density of these bands decreased with increasing cell number. In a Coomassie blue stained gel, the main enriched protein band (69 kDa) as indicated by the lower arrow constitutes about 13.6% of total protein from 10^4^ U251n cells (**Fig. 1C**). Western blotting analysis revealed a cell-density-dependent protein alteration due to the uneven protein loading. It is important to note that beta-actin is easily saturated. Therefore when beta-actin is used as the internal control, the chemoluminescent signal should not be over- exposed (**Fig. 1D**).

**Figure 2:**
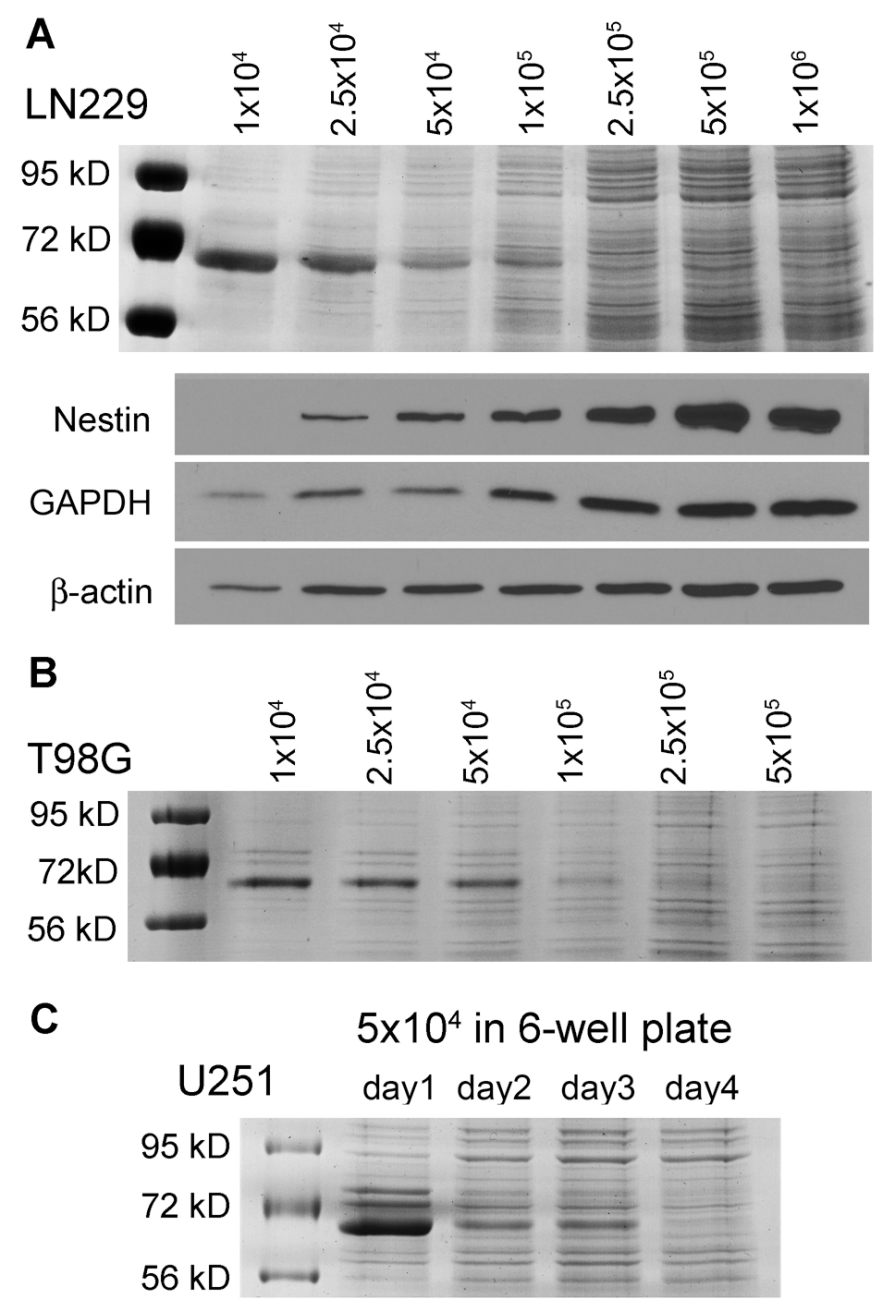
**Enrichment of proteins in different cell lines**. **A**. Coomassie brilliant blue staining of SDS-PAGE gel loaded with LN229 cell proteins. LN229 cells were seeded in 6-well plates at different numbers. 24 h after seeding, total cell protein was extracted by RIPA buffer and 8 µg of proteins were loaded for analysis. Lower part of the picture shows the Western blotting analysis of proteins from LN229 cells. **B**. Coomassie brilliant blue stained SDS-PAGE gel of T98G glioma cell proteins. T98G cells were seeded in 6-well plates at different numbers. Total cell proteins were extracted 24 h after seeding. **C**. SDS-PAGE gel with U251n proteins stained with Coomassie brilliant blue. 5 × 104 U251n cells were seeded in a 6-well plate (Day 0). Total cell proteins were extracted each day from Day 1 to Day 4.

These protein bands were not only present in U251n cell lysate, but the same bands were also observed in the studies using LN229 and T98G glioma cells (**Fig. 2A** and **2B**). These protein bands are obviously present when the cell number is lower than 2.5 × 10^5^ in 6-well plates. The enriched proteins, in part, led to the uneven loading of cell proteins. To verify that these proteins are not produced in the process of cell dissociation, we seeded 5 × 10^4^ U251n cells in a 6-well plate and let them grow for 4 days. Proteins were extracted every day from Day 1 to Day 4. We found that these proteins were not eliminated during the extended cell growth, but closely correlated with cell density (**Fig. 2C**).

**Figure 3:**
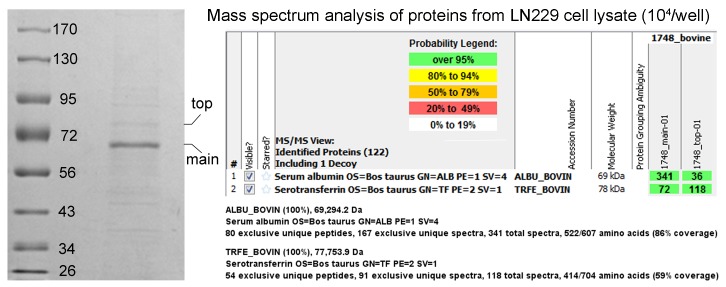
**Mass spectrum analysis of enriched proteins**. Protein samples from LN229 cells were analyzed. Two of the enriched protein bands from SDS-PAGE gel were cut and used for mass spectrum analysis. The proteins identified with the highest scores are shown in the figure.

To identify these proteins, two of the most highly enriched bands from LN229 cell lysate were extracted for mass spectrum (MS) analysis. When matching the MS results with human protein database, we did not find a clear indication of protein. The most likely proteins are heat shock cognate protein (70 kDa) and prelamin-A protein (74 kDa). However, the matching score is less than 70, and our Western blotting analysis did not support heat shock protein (data not shown). Highly matched scores were obtained from bovine protein analysis. The most highly enriched band is bovine serum albumin (69 kDa) and the other band is bovine serotransferrin (78 kDa) (**Fig. 3**).

**Figure 4:**
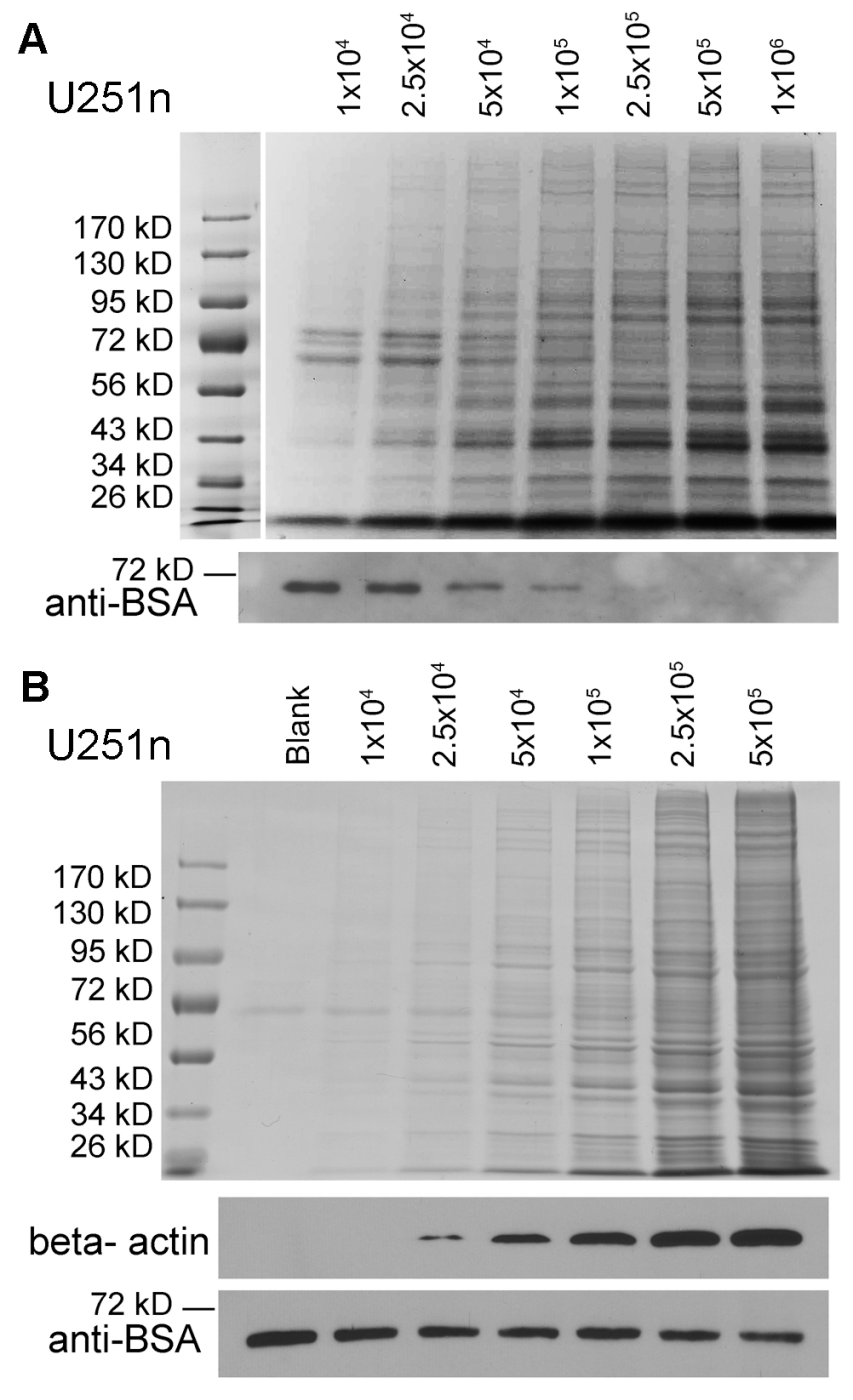
**Detection of bovine serum albumin by Western blotting analysis**. **A**. U251n proteins from increasing numbers of cells in 6-well plates were separated by TCE containing SDS-PAGE and visualized by UV light as introduced in the Methods section. A Western blotting analysis of BSA is shown under the total protein picture. **B**. Different numbers of U251n cells were seeded in a 6-well plate. 24 h later, cells from each well were washed 3 times with ice-cold PBS and total proteins were extracted by 200 µl of RIPA buffer. A blank well without cells was used as control and a 20-µl sample was applied to SDS-PAGE analysis. Total proteins were stained with Coomassie brilliant blue. A Western blotting analysis of BSA is shown under the protein staining.

The serum albumin band was confirmed by Western blotting analysis (**Fig. 4A**). The size and density of the albumin bands was consistent with the results from the SDS-PAGE gel. Thus, we confirmed that these proteins are from cell culture medium. Additionally, we seeded different amounts of U251n cells in 6-well plates and collected cells using the same amount of lysis buffer. The highest amount of serum albumin was collected from blank wells, indicating that cell culture plasticware adsorb serum proteins; the more area that is occupied by cells, the less opportunity for serum proteins to bind to vacant spots on the surface (**Fig. 4B**).

FBS is a natural product and is composed of numerous types of proteins. Proteins such as macromolecular proteins, low molecular weight nutrients, growth factors and other compounds are necessary for *in vitro* growth of cells. Zhang *et al*. used proteomic techniques to analyze the protein composition of the three lots of FBS. The amount of total protein ranged from 3.2 to 4.2 mg/ml [3]. The medium we used in this study contains 4.1 mg/ml protein as detected by BCA method. The major component of FBS is serum albumin which constitutes 60 to 67% of total proteins. Other abundant proteins include cone cGMP-specific 3’,5’-cyclic phosphodiesterase R-subunit, alpha-1-antiproteinase, plasminogen and lactoperoxidase. It was reported that transferrin is relatively constant in serum and ranges from 1.37 mg/ml to 3.72 mg/ml [4, 5]. Serum transferrin is not the most abundant protein in FBS. However, high levels of transferrin were detected in protein extraction from polystyrene coated plasticware. This may be caused by the different affinities of proteins to polystyrene.

**Figure 5:**
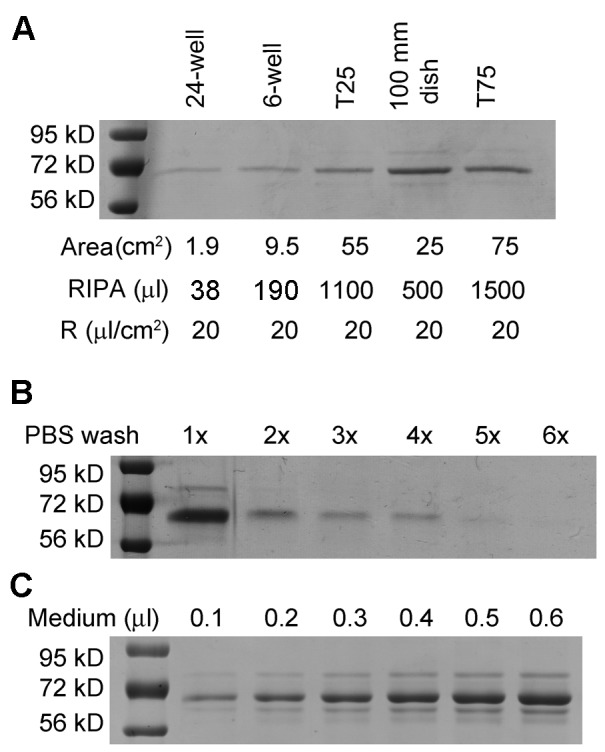
**SDS-PAGE analysis of serum proteins**. Gels were stained with Coomassie brilliant blue. **A**. FBS-containing medium was added into different cell culture plasticware. 24 h later, the plasticware were washed 3 times with ice-cold PBS, then RIPA buffer was applied to extract proteins. The amount of RIPA buffer was used according to the growth areas of each plasticware. 20 µl/cm^2^ of RIPA buffer was used for extraction. 20 µl of extracted protein lysates were applied to SDS-PAGE gels. **B**. 4 ml of FBS-containing medium was added to 6-well plates. 24 h later, 5-ml of ice-cold PBS was used to wash the wells at different times. 200 µl of RIPA buffer was used to extract proteins. 20 µl of protein lysates were applied to SDS-PAGE gels. **C**. Protein analysis of different amounts of FBS containing medium in SDS-PAGE gel. These results were achieved 3 times, and this figure shows a typical result.

Our results showed that washing cells 3 times with PBS could not remove all the serum proteins in a 6-well plate. Further, we tested serum protein adsorption using other types of cell culture plasticware such as 24-well plate, 6-well plate, 100 mm dish, T25 and T75. In this experiment, the volume of RIPA buffer was added according to the surface area of each plasticware. A 20 µl/cm2 RIPA buffer was applied. The results showed that serum proteins could be detected in all of the tested cell culture plasticware. The variation of serum protein collected may be due to the differences in plasticware shape and ease of extraction (**Fig. 5A**). For the 6-well plate, a total 2.3 µg of protein was collected by RIPA buffer after washing 3 times with PBS, which is equal to 0.56 µl of full medium. By using a 6-well plate, we tested for remaining protein after washing with PBS at several different times. The results showed that a trace amount of the serum albumin band was still visible in the Coomassie blue stained SDS-PAGE gel after washing the cells 5 times with PBS (**Fig. 5B**). When running FBS containing cell culture medium in SDS-PAGE gels, more than two protein bands were observed. However, the extracted proteins from plasticware showed two main bands (**Fig. 5C**). Again, this may be due to the different binding affinities of proteins to plasticware.

**Figure 6:**
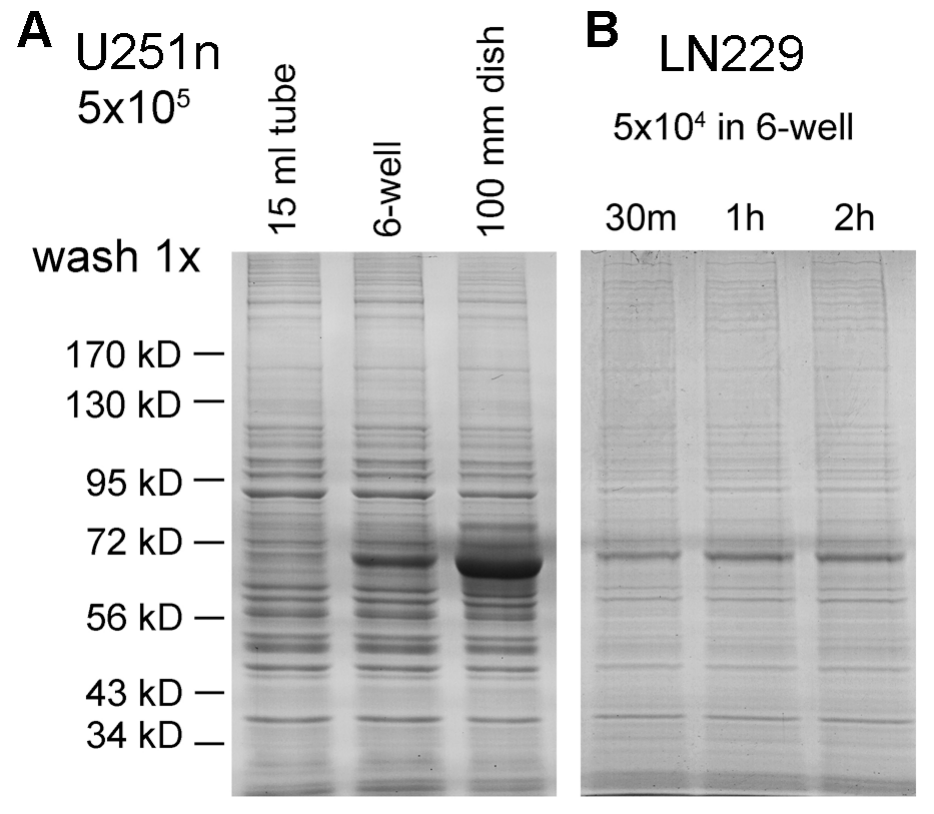
**Verification of serum-protein adsorption by plasticware**. **A**. 5 × 10^5^ U251n cells were seeded in a 6-well plate and 100-mm dish. 24 h later, one set of cells from the 6-well plate and cells from the 100-mm dish were washed once with PBS, and proteins were extracted with RIPA buffer. Another set of cells from the 6-well plate was dissociated with trypsin-EDTA. Cells were collected with cell culture medium and centrifuged down to get cell pellet. Cells were re-suspended and washed once with 10 ml of PBS. Then cell pellet was lysed with RIPA buffer. 8 µg of protein was applied to SDS-PAGE gel and stained with Coomassie brilliant blue. **B**. LN229 cells were seeded in 6-well plate with 5 × 10^4^ in each well. Supernatant with cells was removed and washed with ice-cold PBS at 30 min, and 1- and 2-hr time points. The attached cells were washed with PBS 3 times and lyzed with RIPA buffer. Cells from supernatant were washed and added to the lysate. 8 µg of total protein was applied for SDS-PAGE analysis.

Additionally, we confirmed protein adsorption by extracting proteins from cell pellets. Even a single washing with PBS removed a significant amount of serum protein from dissociated cells (**Fig. 6A**). On the contrary, substantial amounts of serum protein remained in the 6-well plate and 100-mm dish after one washing with PBS, which suggests that serum proteins are adsorbed on cell culture plasticware. We also found that protein adsorption to plasticware occurs quickly and that serum proteins could be extracted as early as 30 min after medium was added in 6-well plates (**Fig. 6B**).

Polystyrene is a commonly used polymer to treat cell culture plasticware. Polystyrene enhances cell attachment as well as adsorption of protein and nuclear acid [6-8]. As indicated by Corning’s technical report, polystyrene-treated surfaces can bind 0.45 µg BSA per square centimeter [9]. Our current results were obtained by using plasticware from a single vendor. Further studies should examine the degree of protein retention in plasticware from a variety of vendors. We recommend that the presence of serum proteins should be considered, if proteins are collected from cell culture plasticware. When serum proteins are suspected in protein samples, whole protein visualization methods such as standard Coomassie blue staining or stain-free techniques should be used to verify the presence of serum albumin. The amount of protein can thus be adjusted by excluding the suspected bands.
